# Clinical and immunological characteristics of vaccinated patients with COVID-19

**DOI:** 10.1097/CM9.0000000000002267

**Published:** 2022-08-18

**Authors:** Yu Ping, Jianmin Huang, Feifei Fan, Yongjun Guo, Jianjun Gou, Yi Zhang

**Affiliations:** 1 Biotherapy Center, The First Affiliated Hospital of Zhengzhou University, Zhengzhou, Henan 450000, China; 2 Department of Respiratory Medicine, The First Affiliated Hospital of Zhengzhou University, Zhengzhou, Henan 450000, China; 3 Department of Pathology, Henan Academy of Medical Sciences, Zhengzhou, Henan 450000, China; 4 Department of Clinical Laboratory, The First Affiliated Hospital of Zhengzhou University, Zhengzhou, Henan 450000, China; 5 State Key Laboratory of Esophageal Cancer Prevention and Treatment, Zhengzhou, Henan 450000, China.

*To the Editor*: Coronavirus disease 2019 (COVID-19), caused by severe acute respiratory syndrome coronavirus 2 (SARS-CoV-2), is a serious public health crisis threatening human health and survival worldwide.^[1]^ Reportedly, laboratory characteristics such as C-reactive protein, lactate dehydrogenase, D-dimer, plasma cytokines, blood platelets, and leukocytes are abnormal in patients with SARS-CoV-2 infections.^[2,3]^ To enhance the protective immune response against SARS-CoV-2, vaccines have been rapidly developed and administered to healthy individuals or patients with various diseases in several countries.^[4,5]^ However, some healthy individuals have still become infected with SARS-CoV-2 despite vaccination. Changes in the clinical and laboratory indices of vaccinated patients with COVID-19 remain unknown. In the present study, we report the effects of vaccination in patients with COVID-19.

A total of 116 hospitalized patients with COVID-19 without any comorbidities or complications were enrolled in the First People's Hospital of Zhengzhou City (Southern District) in Zhengzhou, Henan, in August 2021. All patients tested positive for SARS-CoV-2, as assessed using nucleic acid tests. The enrolled patients excluding asymptomatic patients were divided into three clinical subtypes (2 severe, 52 moderate, and 46 mild) based on the guidelines provided in the Diagnosis and Treatment Protocol for COVID-19 (Trial Version 8) issued by National Health Commission of the People's Republic of China (http://www.nhc.gov.cn/). Supplementary Table 1 presents the basic clinical characteristics of enrolled patients. This study was approved by the Ethics Committee of the First Affiliated Hospital of Zhengzhou University (No. 2021-KY-0728). The requirement to obtain the informed consent was waived.

Among the 116 patients, 62 were vaccinated: four had been vaccinated with a single dose, 57 with two doses, and one with three doses. No differences in clinical subtypes were detected between vaccinated and unvaccinated patients [Supplementary Table 1]. Among the four patients vaccinated with a single dose, one presented with severe disease, two had moderate disease, and one exhibited mild symptoms. In contrast, of the 57 patients who had received two doses of the vaccine, 27 (47.4%) had moderate-intensity disease, 22 (38.6%) exhibited mild manifestations, and eight (14.0%) were asymptomatic.

To further evaluate the clinical significance of vaccination, the clinical and laboratory parameters of patients with COVID-19 were analyzed. Lung computed tomography (CT) images were available in 78 of the enrolled patients with COVID-19. Among the 34 unvaccinated patients with COVID-19, lung CT images of 20 patients (58.8%) showed features of SARS-CoV-2 infection. In contrast, only 19 of the 44 vaccinated patients with COVID-19 (43.2%) exhibited changes in lung images. In addition, the time required from positive SARS-CoV-2 RNA copies to negative results was shorter in vaccinated patients with COVID-19 than in unvaccinated patients (24.50 [19.00–30.00] days *vs.* 30.00 [23.00–40.00] days, *P* = 0.010). Reportedly, IgM antibodies against the SARS-CoV-2 protein indicate an early immune response, and IgG antibodies appear following IgM antibodies, representing the highest opsonization and neutralization activities during the immune response. In our study, we found the IgG to IgM (IgG/IgM) ratio was higher in patients with COVID-19 who had undergone vaccination than in unvaccinated patients (74.01 [14.18–214.78] *vs.* 2.86 [0.80–12.54], *P* < 0.001). Interestingly, we observed that thrombin time in vaccinated patients with COVID-19 was shorter than that in unvaccinated patients (17.42 ± 0.98 s *vs*. 18.06 ± 1.07 s, *P* = 0.004). Cytokine release is an important clinical monitoring index in patients with COVID-19. As 98.3% (114/116) of the enrolled patients had moderate, mild, or asymptomatic disease in the present study, none of the patients displayed characteristics of a severe cytokine storm. Therefore, we analyzed the differences in inflammatory cytokine between vaccinated and unvaccinated patients. Anti-inflammatory cytokines, such as IL-10, showed a downward trend in unvaccinated patients with COVID-19, compared with those in patients with COVID-19 who had been vaccinated; however, the difference was not statistically significant (5.15 [3.93–6.34) pg/mL *vs*. 5.80 [4.20–7.20] pg/mL, *P* = 0.345). Consistent with the observations for anti-inflammatory cytokines, proinflammatory cytokines such as IL-2, tumor necrosis factor (TNF)-α, and IL-12 p70 also exhibited a downward trend in unvaccinated patients with COVID-19, when compared with their vaccinated counterparts (IL-2: 9.05 [7.20–16.30] pg/mL *vs*. 9.30 [7.00–27.10] pg/mL, *P* = 0.457; TNF-α: 2.10 [1.00–4.40] pg/mL *vs*. 2.30 [1.60–7.50] pg/mL, *P* = 0.334; IL-12 p70: 0.80 [0.50–1.60] pg/mL *vs*. 1.25 [0.55–1.80] pg/mL, *P* = 0.078).

The inflammatory cytokine profiles of two patients with severe disease were compared. Proinflammatory (IL-2, IL-6, TNF-α, and IL-12 p70) and anti-inflammatory (IL-4 and IL-10) cytokines showed increasing trends in vaccinated patients as compared with unvaccinated patients. In addition, an unvaccinated patient with a moderate COVID-19 subtype exhibited a mild increase in IL-10, TNF-α, and IL-12 p70 on day 12 of hospitalization; however, a vaccinated patient with moderate COVID-19 displayed relatively high IL-2 and TNF-α on day 15, suggesting that vaccinated patients with COVID-19 are more likely to stimulate an immune response. The CT images of the two patients with moderate COVID-19 revealed SARS-CoV-2 infection in the lungs of the unvaccinated patient. Furthermore, we analyzed immune-related indices in different subtypes with or without vaccination. The IgG/IgM ratio in different subtypes was significantly enhanced after vaccination (moderate: 59.37 [9.34–158.98] *vs*. 1.94 [1.24–2.93], *P* < 0.001; mild: 72.16 [11.79–214.78] *vs*. 2.51 [0.59–11.74], *P* < 0.001; asymptomatic: 362.78 [142.19–559.23] *vs*. 21.99 [6.47–92.40], *P* = 0.003). Cytokine IL-12 p70 significantly increased among those vaccinated patients with moderate-intensity disease (1.20 [0.80–1.50] pg/mL *vs*. 0.60 [0.50–1.00] pg/mL, *P* = 0.025), and most cytokines were elevated in those vaccinated paitents with the mild subtype (IL-2: 12.80 [7.00–29.90] pg/mL *vs*. 7.90 [6.80–15.30] pg/mL, *P* = 0.039; IL-4: 7.80 [5.60–15.60] pg/mL *vs*.7.20 [5.90–11.00] pg/mL, *P* = 0.768; IL-6: 45.30 [43.10–60.00] pg/mL *vs*. 45.10 [42.30–50.30] pg/mL, *P* = 0.376; IL-10: 6.60 [5.30–16.70] pg/mL *vs*. 5.50 [3.93–6.34] pg/mL, *P* = 0.140; IL-12 p70: 1.40 [0.60–1.90] pg/mL *vs*. 0.90 [0.40–1.30] pg/mL, *P* = 0.217). IL-6 exhibited increasing trends in asymptomatic vaccinated patients as compared with unvaccinated patients (43.60 [42.75–68.95] pg/mL *vs*. 42.45 [41.65–44.90] pg/mL, *P* = 0.317). These findings demonstrate that vaccinated patients have better immune responses against SARS-CoV-2 infections than unvaccinated patients.

Furthermore, we analyzed the predictive effect of clinical indicators found in vaccinated patients on time required from detection of positive SARS-CoV-2 RNA copies to negative results. Accordingly, we constructed a receiver operating characteristic (ROC) curve. Based on the time required from detection of positive SARS-CoV-2 RNA copies to negative results (cut-off point = 25 days), vaccinated patients with COVID-19 were divided into two groups. The area under the curve of IL-2, IL-4, IL-6, IL-10, TNF-α, IL-12 p70, IgG/IgM and thrombin time were 0.6665, 0.5454, 0.5681, 0.6092, 0.6494, 0.4750, 0.7470, and 0.5482, respectively, indicating that IgG/IgM might be an ideal predictive factor in vaccinated patients in clinical practice [Figure 1A; Supplementary Table 2]. In addition, ROC curves in unvaccinated patients were generated. IgG/IgM had no effect on clinical outcome prediction; however, thrombin time might be an appropriate indicator in unvaccinated patients [Figure 1B; Supplementary Table 3]. Univariable and multivariable logistic regression analyses were also performed to further improve the accuracy of these predictive indicators [Supplementary Table 4]. Finally, it was identified that the IgG/IgM was considered an independent prognostic indicator in vaccinated patients, whereas thrombin time was not an ideal prognostic indicator in unvaccinated patients.

**Figure 1:**
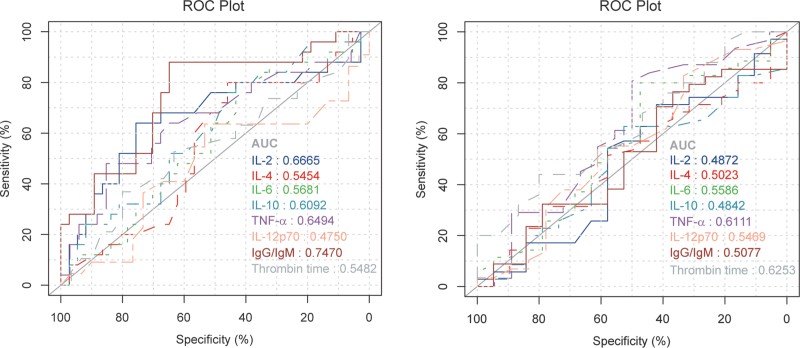
ROC curves of IL-2, IL-4, IL-6, IL-10, TNF-α, IL-12 p70, IgG/IgM and thrombin time in vaccinated patients (A) and unvaccinated patients (B). AUC: Area under the curve; COVID-19: Coronavirus disease 2019; IgG: Immunoglobulin G; IgM: Immunoglobulin M; IL: Interleukin; ROC: Receiver operating characteristic; TNF-α: Tumor necrosis factor-α.

Currently, the impact of vaccination on COVID-19 patients is unclear. However, there are no clear indicators for predicting patient prognosis. Therefore, we identified an ideal indicator to predict the outcome in vaccinated patients, but this does not apply to unvaccinated patients. Further studies are needed to confirm these findings with respect to outcomes in COVID-19 patients.

In summary, we have identified several vaccine-related indicators of COVID-19 patient outcomes (IgG/IgM, thrombin time, and inflammatory cytokine). Furthermore, proinflammatory cytokines (IL-2 and TNF-α) exhibited an upward trend in vaccinated patients during hospitalization. Finally, IgG/IgM was identified as an ideal indicator for predicting clinical outcomes in vaccinated patients.

## Acknowledgments

We thank all the medical staff and physicians fighting against COVID-19 in the First Affiliated Hospital of Zhengzhou University and the First People's Hospital of Zhengzhou City (Southern District).

## Funding

This work was supported by grants from the Emergency Prevention and Control Scientific Research Project of Henan Province (Nos. 211100310700 and 211100310900) and Major Public Welfare Projects in Henan Province (No. 201300310400).

## Conflicts of interest

None.

## Supplementary Material

Supplemental Digital Content
