# Supplementary material for: Clinical and immunological characteristics of vaccinated patients with COVID-19
Source: Chin Med J (Engl). 2022 Aug 18;135(14):1756–8. doi: 10.1097/CM9.0000000000002267 (PMC9509181; doi:10.1097/CM9.0000000000002267)
Supplement: Supplemental Digital Content [file cm9-135-1756-s001.docx]

**Supplementary Table 1: Basic clinical characteristics of 116 COVID-19 patients.**

| **Characteristic** | **Vaccinated (*n*=62)** | **Unvaccinated** **(*n*=54)** | ***Z*/*χ*^2^** | ***P*-value** |
| --- | --- | --- | --- | --- |
| Age (years) | 38.5 (31.0–50.0) | 32.0 (25.0–54.0) | 1.307 | 0.253 |
| Sex |  |  | 0.357 | 0.550 |
| Male | 31 (50.00) | 30 (55.56) |  |  |
| Female | 31 (50.00) | 24 (44.44) |  |  |
| Subtype |  |  | - | 0.470 |
| Severe | 1 (1.61) | 1 (1.85) |  |  |
| Moderate | 30 (48.39) | 22 (40.74) |  |  |
| Mild | 23 (37.10) | 23 (42.59) |  |  |
| Asymptomatic | 8 (12.90) | 8 (14.81) |  |  |

Data are presented as median (range) or *n* (%). COVID-19: Coronavirus disease 2019; -: Not applicable.

**Supplementary Table 2: Parameter information of ROC curve in vaccinated patients with COVID-19.**

| **Index** | **IL-2 (pg/mL)** | **IL-4 (pg/mL)** | **IL-6 (pg/mL)** | **IL-10 (pg/mL)** | **TNF-α (pg/mL)** | **IL-12p70 (pg/mL)** | **IgG/IgM** | **Thrombin time (s)** |
| --- | --- | --- | --- | --- | --- | --- | --- | --- |
| AUC | 0.6665 | 0.5454 | 0.5681 | 0.6092 | 0.6494 | 0.4750 | 0.7470 | 0.5482 |
| Best cut-off value | 12.25 | 7.85 | 43.45 | 4.65 | 6.10 | 1.35 | 95.75 | 16.75 |
| Sensitivity (%) | 64.00 | 80.00 | 80.00 | 80.00 | 48.00 | 63.64 | 88.00 | 36.84 |
| Specificity (%) | 75.68 | 45.95 | 43.24 | 43.24 | 85.29 | 53.33 | 64.86 | 80.00 |
| Negative predictive value (%) | 75.68 | 77.27 | 76.19 | 76.19 | 69.05 | 66.67 | 88.89 | 66.67 |
| Positive predictive value (%) | 64.00 | 50.00 | 48.78 | 48.78 | 70.59 | 50.00 | 62.86 | 53.85 |
| True positive rate (%) | 64.00 | 80.00 | 80.00 | 80.00 | 48.00 | 63.64 | 88.00 | 36.84 |
| False positive rate (%) | 24.32 | 54.05 | 56.76 | 56.76 | 14.71 | 46.67 | 35.14 | 20.00 |
| True negative rate (%) | 75.68 | 45.95 | 43.24 | 43.24 | 85.29 | 53.33 | 64.86 | 80.00 |
| False negative rate (%) | 36.00 | 20.00 | 20.00 | 20.00 | 52.00 | 36.36 | 12.00 | 63.16 |
| False discovery rate (%) | 36.00 | 50.00 | 51.22 | 51.22 | 29.41 | 50.00 | 37.14 | 46.15 |
| Accuracy (%) | 70.97 | 59.68 | 58.06 | 58.06 | 69.49 | 57.69 | 74.19 | 63.27 |
| Precision (%) | 64.00 | 50.00 | 48.78 | 48.78 | 70.59 | 50.00 | 62.86 | 53.85 |
| Youden index | 139.68 | 125.95 | 123.24 | 123.24 | 133.29 | 116.97 | 152.86 | 116.84 |

AUC: Area under the curve; COVID-19: Coronavirus disease 2019; IgG: Immunoglobulin G; IgM: Immunoglobulin M; IL: Interleukin; ROC: Receiver operator characteristic; TNF-α: Tumor necrosis factor-α.

**Supplementary Table 3: Parameter information of ROC curve in unvaccinated patients with COVID-19.**

| **Index** | **IL-2 (pg/mL)** | **IL-4 (pg/mL)** | **IL-6 (pg/mL)** | **IL-10 (pg/mL)** | **TNF-α (pg/mL)** | **IL-12p70 (pg/mL)** | **IgG/IgM** | **Thrombin time (s)** |
| --- | --- | --- | --- | --- | --- | --- | --- | --- |
| AUC | 0.4872 | 0.5023 | 0.5586 | 0.4842 | 0.6111 | 0.5469 | 0.5077 | 0.6253 |
| Best cut-off value | 12.20 | 9.80 | 48.00 | 4.55 | 1.15 | 0.85 | 1.34 | 18.35 |
| Sensitivity (%) | 71.43 | 31.43 | 80.00 | 62.86 | 80.65 | 55.17 | 76.47 | 44.00 |
| Specificity (%) | 42.11 | 78.95 | 47.37 | 52.63 | 50.00 | 61.11 | 36.84 | 80.00 |
| Negative predictive value (%) | 44.44 | 38.46 | 56.25 | 43.48 | 60.00 | 45.83 | 46.67 | 46.15 |
| Positive predictive value (%) | 69.44 | 73.33 | 73.68 | 70.97 | 73.53 | 69.57 | 68.42 | 78.57 |
| True positive rate (%) | 71.43 | 31.43 | 80.00 | 62.86 | 80.65 | 55.17 | 76.47 | 44.00 |
| False positive rate (%) | 57.89 | 21.05 | 52.63 | 47.37 | 50.00 | 38.89 | 63.16 | 20.00 |
| True negative rate (%) | 42.11 | 78.95 | 47.37 | 52.63 | 50.00 | 61.11 | 36.84 | 80.00 |
| False negative rate (%) | 28.57 | 68.57 | 20.00 | 37.14 | 19.35 | 44.83 | 23.53 | 56.00 |
| False discovery rate (%) | 30.56 | 26.67 | 26.32 | 29.03 | 26.47 | 30.43 | 31.58 | 21.43 |
| Accuracy (%) | 61.11 | 48.15 | 68.52 | 59.26 | 69.39 | 57.45 | 62.26 | 57.50 |
| Precision (%) | 69.44 | 73.33 | 73.68 | 70.97 | 73.53 | 69.57 | 68.42 | 78.57 |
| Youden index | 113.53 | 110.38 | 127.37 | 115.49 | 130.65 | 116.28 | 113.31 | 124.00 |

AUC: Area under the curve; COVID-19: Coronavirus disease 2019; IgG: Immunoglobulin G; IgM: Immunoglobulin M; IL: Interleukin; ROC: Receiver operator characteristic; TNF-α: Tumor necrosis factor-α.

**Supplementary Table 4: Univariable and multivariable regression analysis of IgG/IgM and thrombin time.**

| **Characteristics** | **Univariable analysis** | | **Multivariable analysis** | | | |
| --- | --- | --- | --- | --- | --- | --- |
|  | **Model 1** | | **Model 2** | | **Model 3** | |
|  | **OR (95% CI)** | ***P-*value** | **OR (95% CI)** | ***P-*value** | **OR (95% CI)** | ***P-*value** |
| IgG/IgM^*^ | 0.074 (0.019–0.294) | 0.0002 | 0.074 (0.018–0.306) | <0.001 | 0.034 (0.006–0.214) | 0.0003 |
| Thrombin time^†^ | 3.143 (0.707–13.964) | 0.132 | 3.172 (0.644–15.618 | 0.156 | 3.117 (0.606–16.027 | 0.174 |

OR: Odds ratio; CI: Confidence interval. ^*^Predictor of vaccinated patient. ^†^Predictor of unvaccinated patient. Model 1: Unadjusted; Model 2: Adjusted for age and sex; Model 3: Adjusted for age, sex and clinical subtypes.
